# Alendronate decreases orthotopic PC-3 prostate tumor growth and metastasis to prostate-draining lymph nodes in nude mice

**DOI:** 10.1186/1471-2407-8-81

**Published:** 2008-03-28

**Authors:** Johanna M Tuomela, Maija P Valta, Kalervo Väänänen, Pirkko L Härkönen

**Affiliations:** 1 Institute of Biomedicine, Department of Anatomy, University of Turku, Finland; 2 Department of Laboratory Medicine, MAS University Hospital, Lund University, Sweden

## Abstract

**Background:**

Metastatic prostate cancer is associated with a high morbidity and mortality but the spreading mechanisms are still poorly understood. The aminobisphosphonate alendronate, used to reduce bone loss, has also been shown to inhibit the invasion and migration of prostate cancer cells *in vitro*. We used a modified orthotopic PC-3 nude mouse tumor model of human prostate cancer to study whether alendronate affects prostate tumor growth and metastasis.

**Methods:**

PC-3 cells (5 × 10^5^) were implanted in the prostates of nude mice and the mice were treated with alendronate (0.5 mg/kg/day in PBS, s.c.) or vehicle for 4 weeks. After sacrifice, the sizes of tumor-bearing prostates were measured and the tumors and prostate-draining regional iliac and sacral lymph nodes were excised for studies on markers of proliferation, apoptosis, angiogenesis and lymphangiogenesis, using histomorphometry and immunohistochemistry.

**Results:**

Tumor occurrence in the prostate was 73% in the alendronate-treated group and 81% in the control group. Mean tumor size (218 mm^3^, range: 96–485 mm^3^, *n *= 11) in the alendronate-treated mice was 41% of that in the control mice (513 mm^3^, range: 209–1350 mm^3^, *n *= 13) (*p *< 0.05). In the iliac and sacral lymph nodes of alendronate-treated mice, the proportion of metastatic area was only about 10% of that in control mice (*p *< 0.001). Immunohistochemical staining of tumor sections showed that alendronate treatment caused a marked decrease in the number of CD34-positive endothelial cells in tumors (*p *< 0.001) and an increase in that of ISEL positive apoptotic cells in tumors as well as in lymph node metastases (*p *< 0.05) compared with those in the vehicle-treated mice. The density of m-LYVE-1-stained lymphatic capillaries was not changed.

**Conclusion:**

Our results demonstrate that alendronate treatment opposes growth of orthotopic PC-3 tumors and decreases tumor metastasis to prostate-draining lymph nodes. This effect could be at least partly explained by decreased angiogenesis and increased apoptosis. The results suggest that bisphosphonates have anti-tumoral and anti-invasive effects on primary prostate cancer.

## Background

Every year prostate cancer is diagnosed in more than 500 000 men worldwide [1]. Approximately 30 – 50% of them have evidence of metastatic disease, which results in a high morbidity and mortality. In advanced prostate cancer, 25 – 42% of the patients have metastases in regional lymph nodes [2] and about 70% have metastases in bone [3]. The mechanisms behind invasive prostate cancer are still largely unknown and the metastatic disease is basically incurable.

Only a few experimental models of spontaneous prostate cancer exist, since this malignancy is uncommon in nonhuman mammals. To study tumor growth *in vivo*, immunodeficient mice are commonly used as models [4,5]. In athymic nude mice, human prostate cancer cells can be grown subcutaneously or tumor cells can be inoculated orthotopically into the prostate [5]. In order to mimic a metastatic disease, cancer cells can also be inoculated into the circulation either via the tail vein [6] or the left cardiac ventricle [7]. However, considering the structure and function of stroma, vasculature and lymphatic vessels orthotopic tumors provide the most relevant environment for tumor growth and invasion.

Bisphosphonates are used in prevention and treatment of metastatic and myelomatous bone disease [8-10] and osteoporosis [11]. They primarily inhibit osteoclast-mediated bone resorption but in addition to this, they have been shown to have direct effects on different types of tumor cells and tumors. Our previous results [12] showed that the aminobisphosphonate alendronate is able to strongly reduce invasion and migration of PC-3 prostate cancer cells at low, non-toxic concentrations and in a dose-dependent manner. Alendronate also inhibits PC-3 cell adhesion to extracellular matrix proteins in vitro [13,14] and secretion of MMP:s by tumor cells [15]. Other bisphosphonates (clodronate, pamidronate, ibandronate and zoledronate) have been shown to induce apoptosis of breast cancer cells in vitro [16,17], inhibit angiogenesis [18] and, at high concentrations, to inhibit cell proliferation [19].

Interesting results have also been reported in animal models. They show that in models of bone metastasis of breast cancer, bisphosphonates effectively reduce tumor burden in bone [20,21], which has primarily been explained by inhibition of osteoclast-mediated bone resorption and decreased release of bone matrix-bound growth factors. Besides these indirect effects on bone metastasis, bisphosphonates have been shown to decrease tumor spread and formation of visceral metastases and increase survival in tumor-bearing mice. Alendronate has been shown to inhibit intraperitoneal dissemination of ovarian cancer cells [22] and the newer aminobisphosphonates in particular, such as zoledronate and minodronate, have been reported to inhibit growth in 4T1/luc breast tumors [23], melanoma [24], cervical carcinoma [25] and mesothelioma tumors [19], as well as to suppress lung metastasis in osteosarcoma-bearing mice [26]. In addition, zoledronate can inhibit angiogenesis in subcutaneous matrix implants [27], in the prostates of testosterone-treated castrated rats [18] and in a mouse model of cervical carcinogenesis [19].

In clinical trials, Kanis *et al*. [8] and Powles *et al*. [28] showed that clodronate decreased the tumor burden of breast cancer in bone. The overall survival of the patients was better but there was no difference in visceral metastases. In a study by Diel *et al*. [29], clodronate reduced both bone and visceral metastases in breast cancer patients, although Saarto *et al*. [30] have reported the conflicting results as regards visceral metastasis.

The aim of the present study was to examine the effect of alendronate treatment on prostate tumor growth, invasion and lymph node metastasis. For this purpose we improved and exploited the model of orthotopic PC-3 prostate tumors in nude mice. The effects of the aminobisphosphonate alendronate on prostate tumor growth and spread to prostate-draining sacral and iliac lymph nodes were investigated by measuring tumor size and analyzing the tumors by means of histomorphometry and by carrying out immunohistochemical staining of markers of proliferation, angiogenesis, lymphangiogenesis and apoptosis. Using this model we demonstrate that alendronate decreases PC-3 prostate tumor growth and size of metastases in prostate-draining iliac and sacral lymph nodes in nude mice.

## Methods

### Animals

Eight-week-old male athymic nu/nu mice (Harlan Winkelman GmbH, Borchen, Germany) were maintained in a pathogen-free environment, under controlled conditions (20–21°C, 30–60% relative humidity and 12-hour lighting cycle). They were fed with small-animal food pellets (RM3 ESQC, Special Diet Services, Witham, England) and supplied with autoclaved tap water *ad libitum*. Animal welfare was monitored daily, looking for clinical signs, and the animals were weighed twice a week. The animal experiments were carried out according to the European Convention for the Protection of Vertebrate Animals used for Experimental and other Scientific Purposes, and statutes 1076/85 and 1360/90 of The Animal Protection Law in Finland and EU Directive 86/609. The experimental procedures were reviewed by the local Ethics Committee on Animal Experimentation of the University of Turku and approved by the local Provincial State Office of Western Finland.

### Cell culture

The human prostate cancer cell line PC-3 was obtained from the American Tissue Type Culture Collection (Rockville, MD). At near confluence, the cells were harvested and suspended at a concentration of 5 × 10^5^/20 μl in a sterile dye solution consisting of phosphate-buffered saline (PBS; Biochrom AG, Berlin, Germany) with green food color 33022 (0.5 g/ml; Roberts Oy, Turku, Finland). The cells were kept on ice until used for inoculation. Cell viability was 97% or above at the time of inoculation, as determined by trypan blue staining of the cell suspension.

### Orthotopic inoculation into the prostate

Half an hour before inoculation of tumor cells an analgesic drug (Temgesic, 0.3 μg/g, Schering-Plough Nv, Brussels, Belgium) was injected subcutaneously. The mice were anesthetized by means of isofluran inhalation (1.5–3%, air flow 200 ml/min, Univentor 400 anesthesia unit, Univentor Ltd., Zejtun, Malta) and placed in a supine position under a sterile cover. An incision was made 3 mm above the pubic symphysis and the bladder and seminal vesicles were carefully lifted to expose the dorsal prostate. To improve visual control of correct inoculation into the prostate, the cell suspension was supplemented with nontoxic green food color. Then, 5 × 105 cells with the dye, were slowly inoculated into the ventral prostate through the dorsal prostate at a 45° angle, avoiding the urethra. The success of inoculation could be verified by means of the green color. If leakage into the peritoneal cavity or urethra/bladder was observed, the mice were not included in the experiment. Inoculation was performed with a 30 G needle attached to a 25 μl glass syringe (both Hamilton Bonaduz AG, Bonaduz, Switzerland). After inoculation, the abdominal muscle layer was closed with a 4-0 absorbable suture (Bondek plus, polyglycolic acid-coated suture, Genzyme GmbH, Neu-Isenberg, Germany) and the skin with a 4-0 nonabsorbable suture (monofilament, polyamide suture, Genzyme GmbH, Neu-Isenberg, Germany).

### Tumor growth and metastasis to local lymph nodes

In order to study the effects of alendronate on prostate tumor growth and invasion, mice were randomized according to weight into 2 groups. The orthotopic experiments were performed using the total numbers of 15 and 16/group considered to be large enough for the analyses of the statistical significance of the results. Mice in the alendronate group were daily treated with alendronate (0.05 mg/kg s.c., provided by Merck&Co., Inc., Whitehouse Station, NJ) in 100 μl PBS and mice in the control group were injected daily with 100 μl PBS. Alendronate and control treatments were started on the day of orthotopic inoculation of 5 × 10^5 ^PC-3 cells. The mice were sacrificed 4 weeks after inoculation. Prostate (including tumor) size was measured with calipers and the volume calculated according to the method described by Janik et al. [31] as length × width × depth × π/6. The prostate lobes, selected internal organs (lungs, kidneys, adrenal glands, liver and spleen), regional lymph nodes (iliac and sacral), distant lymph nodes (inguinal, sciatic, axillary and brachial), hind limbs and vertebrae were macroscopically examined for the occurrence of tumors, excised and immersed in 4% neutral-buffered formalin. The hind limbs and vertebrae were radiographed and the bone samples were decalcified in 10% EDTA solution for two weeks before further processing.

### Histology and immunohistochemistry

Formalin-fixed tissue samples were embedded in paraffin and 5-μm sections were cut and stained with hematoxylin and eosin (H&E) using standard techniques. The relative areas of necrosis in tumors and the relative area of tumor metastases in iliac and sacral lymph nodes was determined from H&E-stained sections using an analysis program for histomorphometry (AxioVision software, Carl Zeiss Microimaging GmbH, Oberkochen, Germany). The lymph nodes were cut through and 8 to 10 sections/lymph node were analyzed in order to determine metastatic area and the results were expressed as percentage of the total area of the lymph node.

The tumor sections were treated with antibodies: mouse monoclonal anti-LYVE-1 (a gift from Dr. David Jackson) [32], rat monoclonal anti-CD34 (Santa Cruz Biotechnology Inc., Santa Cruz, CA) or mouse monoclonal anti-Ki-67 (Novocastra Laboratories Ltd., Newcastle upon Tyne, UK) o/n at +4°C. The samples were then treated with biotin-labeled goat anti-mouse secondary antibodies (Vector Laboratories, Burlingame, USA), biotin-labeled rabbit anti-rat secondary antibodies (DAKO Denmark A/S, Glostrup, Denmark), or biotin-labeled rabbit anti-mouse secondary antibodies [33], respectively. A mouse-on-mouse kit (Vector Laboratories, Burlingame, CA) was used with Ki-67 antibody staining to inhibit non-specific staining of anti-mouse secondary antibody. The in situ end labeling (ISEL) method was used to detect apoptotic cells. Negative controls (sections of every sample stained without the primary antibody) were used to verify the specificity of staining. The relative numbers of Ki-67- and ISEL-positive cells were counted by means of a 10 × 10 grid using three sections at 500 μm intervals to make sure that the same cell was not counted twice. Altogether 3.000 cells/tumor from 3 different levels were counted. The length of the CD34-positive vessels was counted from the 3 most vascularized fields of each tumor by drawing lines following stained vessels and measuring the length of the lines using AxioVision software (Carl Zeiss Microimaging GmbH, Oberkochen, Germany). The sections of prostate-draining lymph nodes were studied for Ki-67, CD34 and ISEL immunostaining using the same method as described above. The results were blind-tested by three independent analyzers.

### RNA isolation and Northern blot analysis

Total RNA was extracted from PC-3 cells using the guanidinium isothiocyanate method Chomczynski and Sacchi [34]. Subsequently, 20 μg of RNA was separated by electrophoresis, stained with ethidium bromide and blotted on a GeneScreen Plus nylon membrane (NEN Research Products, Boston, MA), using standard conditions suggested by the manufacturer. A cDNA insert (kindly provided by Professor Kari Alitalo, University of Helsinki, Finland) [35] of human VEGF-C was [^32^P]-dCTP-labeled by the random priming method (Ready-to-go DNA labeling Beads, Pharmacia Biotech, Piscataway, NJ). Hybridization and exposure to X-ray film were carried out using conditions suggested by the manufacturer.

### Western blot analysis

Serum-free DMEM conditioned by PC-3 cells for 2 days was harvested from the cultures. The conditioned medium was centrifuged to remove cell debris. Heparin-binding proteins were isolated from the supernatant with 100 μl of heparin-sepharose (1:1 slurry, Amersham Pharmacia Biotech., Piscataway, NJ) overnight at +4°C. Heparin-sepharose beads were sedimented by centrifugation and washed three times with 20 mM Tris-HCl, pH 7.4, 300 mM NaCl. Heparin-sepharose-bound proteins were extracted by means of 5-min incubation in Laemmli sample buffer at +95°C and separated by sodium dodecyl sulfate-polyacrylamide gel electrophoresis (SDS-PAGE). Medium conditioned by 3 × 10^6 ^cells was used per well. After transfer to nitrocellulose membranes (Bio-Rad), VEGF-C was detected using a 1:500 dilution of a goat polyclonal antihuman VEGF-C antibody (Santa Cruz Biotechnology, USA) and a secondary antibody, horseradish peroxidase-labeled anti-goat IgG (DAKO, Denmark), which was used at 1:2000 dilution. Protein bands were visualized using the ECL chemiluminescence detection system (Amersham Corp.). Conditioned media from human breast cancer cells (MCF-7 cells; 1 × 10^6^/well), stably transfected with VEGF-C, were used as positive controls and normal goat IgG (R&B-Systems Inc., Minneapolis, MN) was used instead of anti-VEGF-C antibody as a negative control.

### Statistics

Results were expressed as mean value ± SD. Statistical significance was taken to be *p *< 0.05, using two-tailed Student's *t*-test.

## Results

### Effects of alendronate on tumor size and lymph node metastasis of orthotopic PC-3 prostate tumors

**Figure 1:**
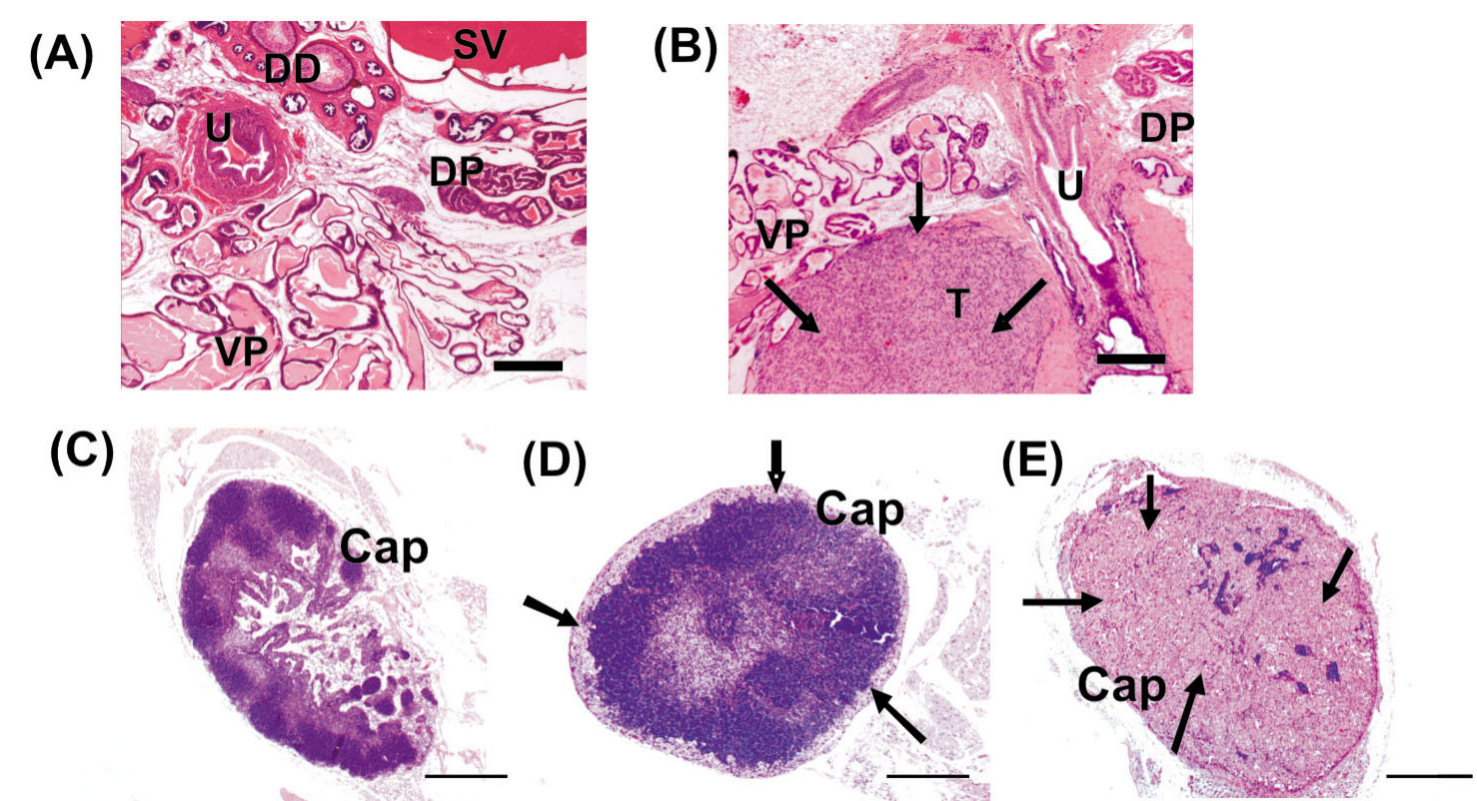
**Morphology of orthotopic PC-3 prostate tumors and metastases in prostate draining lymph nodes**. Prostate from sham-operated control mice (A) and tumor bearing mice (B) 4 weeks after inoculation of PC-3 cells (tumor tissue, arrows). DD = ductus deferens, DP = dorsal prostate, SV = seminal vesicle, T = tumor tissue, U = urethra, VP = ventral prostate (Bar = 500 μm). Prostate draining lymph nodes of sham-operated control mice (C) and from prostate tumor bearing mice (D and E). Infiltrating tumor cells were first observed in the sub-capsular areas of lymph nodes (arrows, D) and in advantaged cases cortex and medulla (arrows, E). Cap = lymph node capsule, (Bar = 500 μm).

Our earlier experiments indicated that PC-3 prostate cancer cells inoculated orthotopically to nude mouse ventral prostate (Fig 1A) form tumors (Fig 1B) and develop lymph node metastases (Figure 1C, D, E) in a time-dependent manner (data not shown). Our refinement of the inoculation method – adding dye to the cell suspension – strengthens the applicability of orthotopic PC-3 tumors and provides a model for studying prostate cancer growth and spread. In successful inoculation no leakage to the peritoneal cavity was observed. The color was first seen in the ventral prostate, from where it spread to the dorsolateral lobes. Spreading of dye was fast, probably partly in a retrograde manner via prostatic ducts and possibly because mouse prostatic lobes are not separated by a capsule, but only by a thin, delicate layer of fibromuscular stroma [4]. Tumors formed in both the ventral and dorsolateral lobes of the prostate (Figure 1B). In the dorsolateral lobes, the gene expression signature is most like that in the peripheral zone of the human prostate [36].

To study the effect of alendronate on tumor growth and spread, nude mice were inoculated orthotopically with 5 × 10^5 ^cells into the ventral prostate and the animals were treated daily either with a subcutaneous injection of alendronate (0.5 mg/kg/day in PBS) (n = 15) or PBS (control, n = 16) for 4 weeks. The dose of alendronate given to the mice was that to be equal to the standard clinical dose. Tumor growth in the prostate was observed in most of the nude mice inoculated with PC-3 cells. The end point of 4 weeks was selected on the basis of previous time course studies (not shown) to ensure that the orthotopic tumors would not grow too big.

Tumors grown in the prostates of nude mice showed a solid and homogeneous morphology in H&E staining (Figure 1A: sham-operated mouse, 1B: mouse inoculated with PC-3 cells). Four weeks after inoculation of PC-3 cells, the tumors were mostly confined to the ventral prostate, in which only a few glandular structures were left, but often tumor cells had spread to all lobes of the prostate gland. Tissue samples that were classified as tumor-negative were re-sectioned and sections from several levels of tissue were stained and analyzed. If samples were classified as tumor-negative twice by three independent analyzers they were considered as being so. Metastasis in prostate-draining lymph nodes was first observed as growth of tumor cells in the sub-capsular area, which later extended to the cortex and medulla. In some mice, the lymph nodes were totally occupied by metastasized tumor (Figure 1 lymph nodes of sham-operated (C) and orthotopic PC-3 tumor bearing mice, D, E).

Based on macroscopic and histological analysis, tumor occurrence was 73% at 4 weeks in the alendronate-treated group and 81% in the PBS-treated group (Table 1). At this 4-week time point metastases were found in 64% of regional iliac and sacral lymph nodes in the group that received alendronate. In the mice treated with PBS, metastasis in iliac and sacral nodes was found in 54% of the animals. The difference between the groups was not statistically significant (Table 1). The treatments did not affect the body weight of the animals (data not shown).

**Table 1:** Occurrence of tumor growth in prostate and lymph node metastasis of in nude mice treated with alendronate (ALN) or saline (PBS)

Treatment	Occurrence of tumor growth in prostate (%)	Occurrence of metastasis in prostate draining lymph nodes (%)
ALN	11/15 (73)	7/11 (64)
PBS	13/16 (81)	7/13 (54)

Tumors were produced by orthotopic inoculation of 5 × 10^5 ^PC-3 cells and tumors were allowed to grow for 4 weeks.

**Figure 2:**
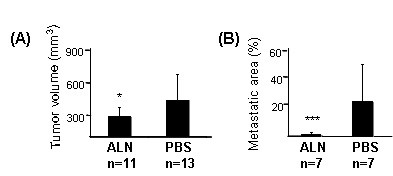
**Effect of alendronate treatment on the size and lymph node metastasis of orthotopic PC3 prostate tumors**. Tumor size was measured using the formula: length × width × depth × π/6. The tumor volume was significantly smaller in alendronate treated mice compared with the control mice (A), p < 0.05, Student's t-test. According to the histomorphometrical analysis (B), the relative area of lymph node metastasis (%) was significantly smaller in alendronate-treated (n = 7) than in PBS-treated control animals (n = 7), p < 0.001, Student's t-test].

The volume of the tumor-bearing prostate at 4 weeks was significantly greater in the control group than in the alendronate-treated group (mean of 513 ± 341 mm^3 ^[range 209–1350 mm^3^] *vs*. mean of 218 ± 123 mm^3 ^[range 96–485 mm^3^], respectively, *p *< 0.05, Figure 2A). Histomorphometric analysis of the lymph nodes also demonstrated that there were marked differences in metastatic invasion of the lymph nodes in alendronate-treated mice compared with control mice. Quantification of the relative areas of metastases showed that the proportion of tumor area in iliac and sacral lymph nodes was significantly smaller in the alendronate group than in the control group (Figure 2B; 2% [ALN] *vs*. 26% [PBS] *p *< 0.001). However, no statistically significant difference was observed in the total size of lymph nodes between treatment groups or between non-metastatic and metastatic lymph nodes at 4 weeks after inoculation of tumor cells. This is possibly caused by several other factors than metastasis that can influence the size and activity of lymph node tissue. This may partly cover the changes caused by metastases. Hence, in addition to macroscopic examination histological examination is always needed in detection of metastasis in lymph nodes.

### Effects of alendronate on morphology and on markers of angiogenesis, proliferation and apoptosis in PC-3 prostate tumors and prostate-draining lymph nodes

**Figure 3:**
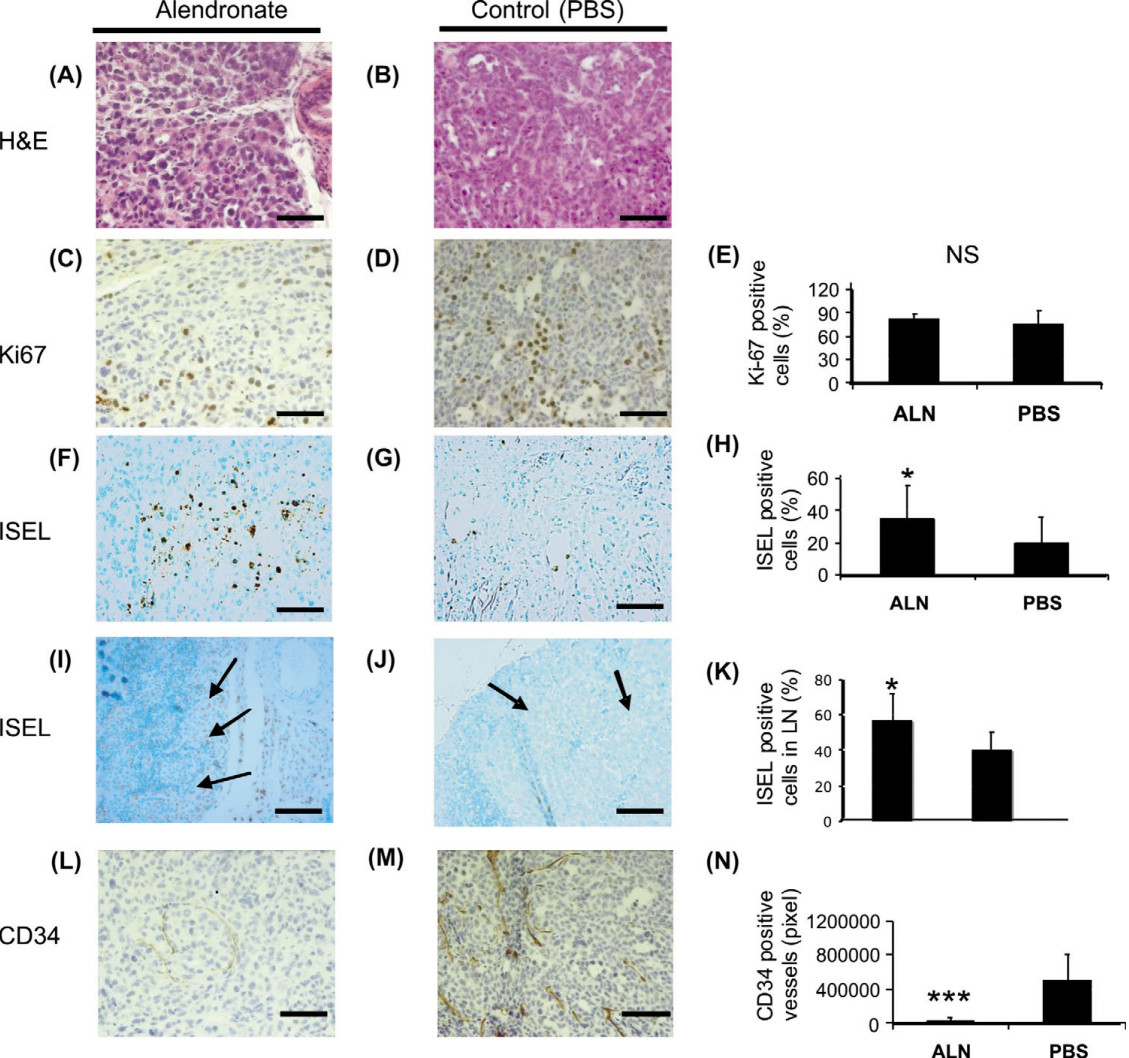
**Effects of alendronate treatment on morphology and on immunohistochemical staining of markers of proliferation (Ki-67), apoptosis (ISEL) and angiogenesis (CD34) in orthotopic PC-3 prostate tumors and lymph nodes**. Alendronate treatment increased apoptosis in tumors and prostate draining lymph nodes and decreased angiogenesis in tumors (Bar = 100 μm). * Significantly different from PBS-treated control, *p *< 0.05, *** significantly different from PBS-treated control, *p *< 0.001, NS nonsignificant (Student's *t*-test).

No changes in morphology were observed in histological (H&E) staining of the prostate tumors in the alendronate and control groups (Figure 3A, B). To assess the characteristics of the tumors in more detail, immunohistochemical staining was performed. The proliferation rate was determined by immunohistochemical staining for Ki-67. No differences were seen between the alendronate group and the control group (Figure 3C–E). The relative number of apoptotic cells was determined by using ISEL staining. Apoptosis was increased by 40% in alendronate-treated tumors in comparison with control tumors (*p *< 0.05, Figure 3F–H). The size of necrotic areas was also increased, but the difference was not statistically significant (data not shown). The prostate draining lymph nodes were also studied by means of immunohistochemistry. Our results demonstrate, that apoptosis was increased by 30% in metastatic tissue of iliac and sacral lymph nodes in alendronate-treated mice compared with vehicle-treated mice (*p *< 0.05, Figure 3L–N). No statistically significant differences were detected in proliferation in metastatic tissue (data not shown). The blood capillaries of orthotopic PC-3 tumors were identified using CD34 antibodies specific for endothelial cells. Capillary density was significantly lower in the alendronate-treated group than in the control group (*p *< 0.001, Figure 3L–N). No statistically significant differences were found, when vascularization of lymph node metastases was studied. On the other hand, the small sizes of the metastatic areas in alendronate-treated lymph nodes made reliable estimation of CD34 staining difficult (data not shown).

### Alendronate treatment has no effect on lymphangiogenesis in PC-3 prostate tumors

**Figure 4:**
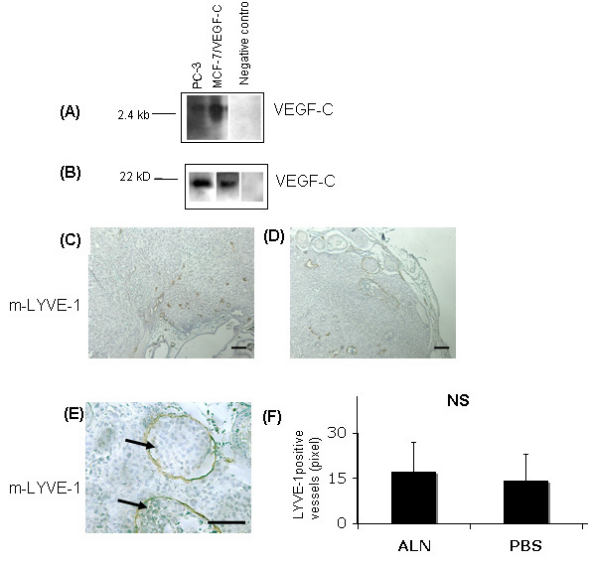
**Network and density of lymphatic vessels in PC-3 prostate tumors from alendronate-treated and PBS-treated mice**. PC-3 cells were analyzed by Northern and Western blots for expression of VEGF-C (A, B, respectively, from left to right: culture media from PC-3 cells, positive control [culture media from MCF-7 cells stably transfected with VEGF-C; n = 1 × 10^6 ^cells], negative control [normal goat IgG]). Sections from PC-3 prostate tumors were immunostained for lymphatic endothelial cells using m-LYVE-1 antibodies (C, D, E). Positive staining was observed primarily in the tumor periphery and in the peritumoral area. Tumor cells were observed also inside lymphatic vessels (E, arrows). Densities of LYVE-1-positive vessels were analyzed from the 3 best vascularized fields/tumor *n *(ALN) = 11, *n *(PBS) = 13. No statistically significant difference was detected between alendronate-treated and control tumors (F, *p *= 0.67, Student's *t*-test). (Bar = 100 μm).

Because alendronate treatment strongly decreased the metastatic area in lymph nodes of PC-3 tumor-bearing mice, we studied the effect of the treatment on the density of lymphatic vessels in tumors. The PC-3 cells were found to produce the lymphangiogenic growth factor VEGF-C, as determined by Northern (Figure 4A) and Western blotting (Figure 4B). This is in accordance with previous results [37,38]. Immunostaining of orthotopic tumors with a mouse-LYVE-1 antibody specific for lymphatic endothelium showed a rich network of lymphatic vessels. In the tumors grown for 4 weeks, staining was mainly observed in the peripheral area of the tumors and in the peritumoral area (Figure 4C [ALN] and 3D [PBS]). In many tumors, tumor cell growth was also observed inside lymphatic capillaries in all treatment groups (Figure 4E, arrows). Alendronate treatment did not, however, result in any differences in the density of mouse-LYVE-1-stained lymphatic capillaries in comparison with vehicle-treated mice (Figure 4F).

### Histological examination of internal organs, long bones and vertebrae for metastases

Only one mouse in the control group had metastatic growth in the lungs. No metastases were found in the liver, spleen or parenchyma of the kidneys or adrenal glands within the time period of the experiment. No bone metastases were found in X-ray analysis or histology of vertebrae, tibiae or femora of mice in any treatment group. The 4-week observation period was probably too short a time to obtain distant metastases using parental PC-3 cells. Our earlier experiments showed that distant metastases in nude mice can be obtained using an intracardiac inoculation method (data not shown) where sufficient tumor cells can be inoculated into the blood stream.

## Discussion

In prostate cancer, 25–42% of patients have metastases in regional lymph nodes at the time of diagnosis [2,39]. Our modified orthotopic PC-3 tumor model, which invades to lymph nodes [5], enables studies on this stage of prostate cancer metastasis. On the other hand, prostate cancer patients often have bone metastases and they are treated with bisphosphonates. Alendronate and other bisphosphonates have been reported to decrease the spread of various other tumors *in vivo*. Our own experiments have also previously shown alendronate inhibition of prostate cancer cell invasion *in vitro*. This prompted us to study the *in vivo *growth and invasion of orthotopic prostate tumors modeling primary prostate cancer.

Alendronate is an aminobisphosphonate that is principally used in treatment of osteoporosis. Detailed molecular mechanisms have not been studied until recently. Alendronate has been shown to have inhibitory effects on osteoclast generation and maturation as well as on osteoclast activity [40]. Although bone metastases of prostate cancer are usually more osteosclerotic than osteolytic, there are indications that bisphosphonates relieve bone pain and inhibit fractures in prostate cancer patients [41-44]. Bisphosphonates thus seem to be beneficial in treatment of prostate cancer bone metastases, at least in subgroups with severe bone pain [44]. In experimental models bisphosphonates have been shown to inhibit tumor invasion when used as a combination therapy with chemotherapeutics (taxol) [45].

Our present results demonstrate that alendronate is able to inhibit prostate tumor growth and invasion to local lymph nodes *in vivo *in nude mice. Surprisingly, a major effect of alendronate, besides being associated with a decreased size of lymph node metastases, was a decrease of tumor size. To our knowledge, alendronate has not earlier been reported to inhibit the growth of primary prostate cancer *in vivo*. Alendronate treatment did not, however, decrease the rate of proliferation as shown by Ki-67 immunohistochemical staining of alendronate *vs*. control-treated prostate tumors. This is in line with the results of our previous studies *in vitro*, in which alendronate did not affect the proliferation of PC-3 cells over a wide range of concentrations that strongly inhibited invasion [12]. Other studies have, however, revealed a decreased rate of proliferation *in vitro *in the presence of alendronate but in those studies very high (micromolar) concentrations of alendronate were used [19,46,47].

In our *in vivo *study alendronate treatment did, however, increase apoptosis in prostate tumors, which could contribute to decreased tumor growth. This is in agreement with the results of some other studies in which *in vitro *cultures [16,17] and *in vivo *tumor models [48] have been used. Relatively high concentrations of bisphosphonates have usually been needed to trigger apoptosis *in vitro *but *in vivo *bisphosphonate treatments with doses and schedules comparable to those used in clinical treatments have been reported to induce apoptosis in several models as regards both bone [20] and visceral metastases [19,22-26]. This suggests that besides direct cellular effects, other mechanisms may also contribute to decreased cell survival *in vivo*.

Another effect that may contribute to decreased tumor size and tumor cell survival in alendronate-treated mice is inhibition of angiogenesis, as suggested by a decreased density of CD34-positive blood vessel capillaries. This observation is also in accordance with earlier reports on the capacity of various bisphosphonates to inhibit angiogenesis *in vitro *and *in vivo *in the context of some physiological and pathological conditions. The decreased angiogenesis associated with bisphosphonate treatment has been related to modulation of endothelial cell proliferation, adhesion, migration [49] and apoptosis [19]. Zoledronate as well as alendronate has been found to inhibit secretion and activity of MMP-2 and MMP-9 by tumor cells and tumor-infiltrating macrophages in mouse models of cervical cancer [19] and prostate cancer [50]. In addition, both minodronate and zoledronate affect VEGF interaction with VEGF receptors and signaling in endothelial cells [19,23]. In our prostate tumors alendronate facilitated apoptosis and inhibited angiogenesis, the latter leading to restricted nutrition and oxygenation, thereby affecting tumor growth. There was, however, no statistically significant increase in the proportion of necrotic areas in alendronate-treated tumors (data not shown).

Orthotopic PC-3 tumors metastasize to sacral and iliac lymph nodes that drain the prostate [5]. Immunostaining for LYVE-1 demonstrated a dense network of lymphatic capillaries in the periphery of tumors as well as in the peritumoral area in our PC-3 prostate tumors. A high level of expression of lymphangiogenic VEGF-C has been shown to correlate with the density of lymphatic capillaries [49,51-53] and increased lymphangiogenesis has been shown to facilitate tumor metastasis to lymph nodes [51-55] although conflicting results have also been published [56]. Our previous studies with orthotopic mammary tumors in nude mice, and those of others, have demonstrated that VEGF-C stimulates lymphangiogenic tumor spread *in vivo *[54,55]. High expression level of VEGF-C has been detected in about 50% of human cancers [49]. PC-3 cells and tumors express VEGF-C at a high level [35,37,38], and it is thus conceivable that the spread of orthotopic PC-3 tumors to lymph nodes occurs primarily via lymphatic vessels. Demonstration of tumor growth inside lymphatic capillaries in our orthotopic tumors also speaks for a lymphatic route of metastasis. Treatment with alendronate reduced the metastatic area in lymph nodes but it did not, however, affect the density of the lymphatic vessel network or the number of lymph node metastases. Therefore, it is possible that the decreased metastatic growth in lymph nodes is explained by alendronate regulation of tumor growth via effects on tumor cells themselves or on angiogenesis, rather than via effects on lymphangiogenesis [18,49]. According to our results increased apoptosis of tumor cells and/or endothelial cells in both primary tumors and lymph node metastases could be a major mechanism by which alendronate decreased metastatic area in iliac and sacral lymph nodes. Our previous results demonstrated a strong inhibitory effect of alendronate on PC-3 cell invasion *in vitro *[12] and other investigations have shown inhibition of MMP secretion and associated invasion capacity of prostate cancer cells [15]. These results are in line with the decreased cell invasion to and limited growth of PC-3 tumors in local lymph nodes of alendronate-treated mice. Decreased angiogenesis is also associated with decreased metastatic potential [57] and could as such contribute to the decreased metastatic growth in lymph nodes.

Our results were based on analyses at an end-point of 4 weeks, which limits the estimation of alendronate effects on tumor formation and progression to this time point. Future experiments with noninvasive imaging methods will allow following growth and spreading of orthotopic tumors at several intervening time points over an optimal time period without markedly expanding the numbers of experimental animals.

There is evidence that the inhibitory effects of aminobisphosphonates are largely mediated via the mevalonate pathway [12,13,40]. Although the precise mode of action of aminobisphosphonates is not understood, the mechanisms that have been recognized are based on their ability to impair post-translational prenylation of Ras, Rac and Rho [12,58]. Aminobisphosphonates have been found to target farnesylpyrophosphate and/or geranylgeranylpyrophosphate synthetase, which leads to decreased generation of farnesyl diphosphate and geranylgeranyl diphosphate. These intermediates are needed for post-translational prenylation of small GTP-binding proteins (Ras, Rac and Rho) which are essential for many cellular functions such as proliferation, survival and invasiveness. It is probable that interference with prenylation reactions is involved in many effects of aminobisphosphonates such as decreased angiogenesis and cell migration as well as increased apoptosis [58].

Recent reports have also shown evidence that aminobisphosphonates can induce apoptosis and/or cause growth arrest to G2/M phase in the same way than pyrophosphate resembling bisphosphonates by production of an endogenous ATP analog [59]. Especially newer aminobisphosphonates can directly inhibit proliferation [19,60]. However, these growth inhibitory effects can be overcome with excess geranylgeranyl pyrophosphate, which links this mechanism to the bisphosphonate inhibition of the mevalonate pathway.

Both aminobisphosphonates and pyrophosphate resembling bisphosphonates have also shown to increase resistance to the apoptotic and growth inhibitory effects in some cancer cells. These effects are associated with activation of p38 mitogen activated protein kinase pathway and may support cell survival and promote proliferation. Interestingly, the p38-mediated effects may not be dependent on the inhibition of the mevalonate pathway [61]. It is possible that the p38-mediated survival effects balance the growth inhibitory effects in the tumor models as ours in which alendronate does not inhibit cell proliferation.

Bisphosphonates bind to calcified bone matrix and accumulate in bone very rapidly, which keeps blood levels of the drug low *in vivo*. Studies carried out by Fournier *et al*. [18] have revealed, however, that in rats at least, some bisphosphonates including clodronate, zoledronate and ibandronate, transiently accumulate in the prostate rather than in several other non-calcified tissues before being trapped by bone. Prostatic levels of bisphosphonates reached a peak at 30–60 minutes after administration of the drug and then declined. The mechanism of bisphosphonate accumulation in tumors is not understood. The results of some studies have suggested that tumor calcification, necrosis or activated macrophages have a role in the uptake process [58,61-64]. Preferential prostatic accumulation may partly explain the anticancer effects of alendronate on prostatic tumors, and increase the possibilities of exploiting bisphosphonates in the development of therapies against prostate cancer.

## Conclusion

The orthotopic PC-3 tumor model mimics the growth of primary prostate cancer and its invasion to lymph nodes. Our study demonstrates that alendronate inhibits orthotopic prostate tumor growth and decreases the size of metastases in lymph nodes *in vivo *in nude mice. Alendronate treatment was associated with strongly inhibited angiogenesis and increased apoptosis, which may largely explain decreased tumor growth. Reduced PC-3 prostate tumor growth together with the previously observed inhibition of PC-3 cell invasion/migration may thus markedly contribute to decreased lymph node metastasis in alendronate-treated mice. The results suggest that besides decreasing metastasis, bisphosphonates can inhibit primary tumor growth in prostate cancer.

## Abbreviations

ALN alendronate, cDNA complementary DNA, DMEM Dulbecco's modified Eagle's medium, DNA deoxyribonucleic acid, EDTA ethylenediaminetetra-acetic acid, H&E hematoxylin-eosine, PBS phosphate-buffered saline, RNA ribonucleic acid, s.c. subcutaneous, VEGF-C vascular endothelial growth factor C.

## Competing interests

The author(s) declare that they have no competing interests.

## Authors' contributions

JT carried out the cell cultures, RNA isolation, Northern Blot and Western Blot analyses, analysis of morphology and morphometry and statistical analysis. Orthotopic tumor experiments were done by JT and MV. PH and KV participated in the design of the study. JT wrote the first version of the manuscript and all authors helped to process it. All authors have read and approved the final manuscript. PH gave final approval for the manuscript to be submitted.

## Pre-publication history

The pre-publication history for this paper can be accessed here:


